# Polyarteritis nodosa initially presenting as concomitant peripheral neuropathy and myositis in unilateral limb: A case report

**DOI:** 10.1097/MD.0000000000034335

**Published:** 2023-07-21

**Authors:** In Jun Han, Chang Hyeon Jeong, Hyoseon Choi

**Affiliations:** a Department of Rehabilitation Medicine, Nowon Eulji Medical Center, Eulji University School of Medicine, Seoul, Republic of Korea; b Department of Rehabilitation Medicine, Yonsei University College of Medicine, Seoul, Republic of Korea.

**Keywords:** case report, myositis, peripheral neuropathy, polyarteritis nodosa

## Abstract

We report the case of a patient who initially presented with peripheral neuropathy and myositis without typical organ involvement, such as the kidneys, skin, or gastrointestinal system, but was ultimately diagnosed with polyarteritis nodosa (PAN).

**Patient concerns:**

A 62-year-old man presented with radicular pain in his right lower extremity. One week later, he complained of right ankle motor weakness and pain in the right posterior thigh, which led to admission. After 6 weeks of hospitalization, he newly experienced pain in his right testicle and anterior thigh.

**Diagnosis:**

The patient was initially diagnosed with polymyositis combined with sciatic neuropathy using magnetic resonance imaging, electrodiagnostic tests, and muscle biopsy. However, with the emergence of other systemic symptoms such as testicular pain, vasculitis was suspected, and the patient was reclassified as PAN using the 2007 European Medicines Agency algorithm and the American College of Rheumatology criteria.

**Interventions:**

The patient was treated with glucocorticoids for more than 6 months, and antiviral medication was prescribed to prevent hepatitis B virus reactivation.

**Outcomes:**

The patient’s radicular pain and pain in the right anterior and posterior thighs and testicle improved, and there were no signs of recurrence.

**Lessons:**

In patients presenting with radicular and focal muscle pain, it is crucial to consider the potential for PAN, as observed in this case report.

## 1. Introduction

Polyarteritis nodosa (PAN) is a rare disease that causes necrosis of medium or small-sized arteries and is not associated with antineutrophil cytoplasmic antibodies (ANCAs).^[1]^ Common symptoms of PAN include loss of appetite and weight, abdominal pain, arthralgia, myalgia, and fever. The symptoms of PAN vary depending on the affected organ. When PAN affects the peripheral nervous system, the most common neurological manifestations are mononeuritis multiplex and symmetrical polyneuropathy.^[2]^ When the muscular system is involved in PAN, it typically presents with myalgia throughout the body; in rare cases, PAN may result in myositis.^[3]^ Most PAN cases presenting as myositis exhibit a bilateral pattern,^[3–5]^ and it is uncommon for myositis to be limited to only one lower extremity. Additionally, PAN cases in which only neurological and muscular symptoms appear, without symptoms in other organs, have rarely been reported. This report describes a rare case of PAN in which the patient initially exhibited peripheral neuropathy and myositis solely in the right lower extremity.

This study was approved by our Institutional Review Board and the requirement for written informed consent was waived (EMCS 2023-04-025).

## 2. Case presentation

A 62-year-old man with a history of hepatitis B virus (HBV) infection visited an outpatient clinic with radicular pain in his right lower extremity and mild lower back pain. He had undergone nerve root block at L4–L5 for diagnostic and therapeutic purposes. After 1 week, his radicular pain persisted, and he presented with newly developed motor weakness in the right ankle and severe muscle pain in the posterior thigh. He complained of a loss of appetite but did not complain of any symptoms related to other specific organs. Manual muscle testing was normal except for ankle dorsiflexion, plantar flexion, and great toe dorsiflexion, which were graded as zero. There was no swelling, palpable mass, or skin lesions in the lower extremities; however, tenderness was noted in the right hamstring muscle.

The initial laboratory results showed white blood cells, alanine aminotransferase, blood urea nitrogen, creatinine, C-reactive protein, erythrocyte sedimentation rate, and creatine phosphokinase levels of 12,440 cells/mm^3^ (4000–10,000), 101 U/L (<40), 13.1 mg/dL (6–20), 0.95 mg/dL (0.7–1.3), 0.76 mg/dL (<0.3), 18 mm/h (<20), and 4482 U/L (46–171), respectively. Gross hematuria was not observed, and urinalysis revealed no evidence of proteinuria or microscopic hematuria. Subsequent laboratory data showed that antinuclear antibody, anti-Jo-1 antibody, cyroglobulin, and ANCA levels were all negative and within the normal range of complement (C3 and C4). The hepatitis C and HIV serology test results were negative. Hepatitis B surface antigen (HBs Ag) in the serum was positive, whereas HBe Ag and HBV DNA were not detected, indicating a non-replicative phase of hepatitis B infection.

**Figure 1.**
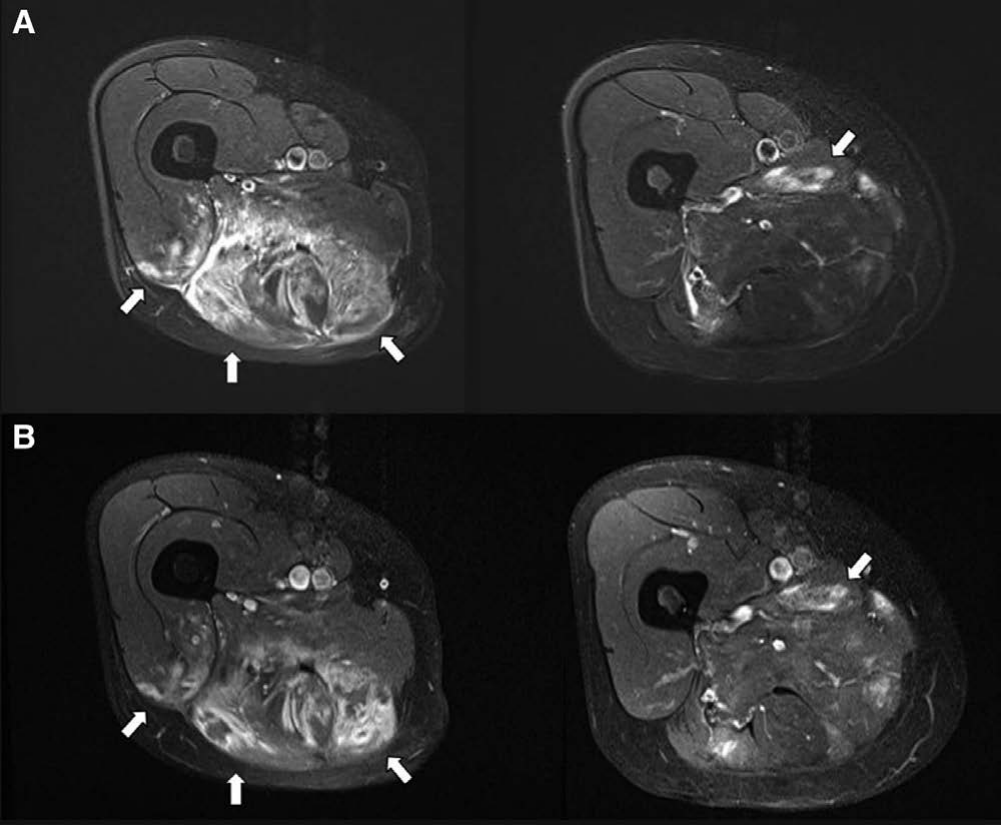
Initial MR images of thigh in a patient with polyarteritis nodosa. (A) Axial fat-suppressed T2-weighted images show diffuse high signal intensity in the biceps femoris, semitendinosus, semimembranosus, and partial involvement in vastus lateralis and vastus intermedius (arrows, left image). High signal intensity is also observed in adductor longus and adductor brevis muscles (arrow, right image). (B) Axial contrast-enhanced fat-suppressed T1-weighted images show diffuse enhancement of the affected muscles (arrows).

**Figure 2.**
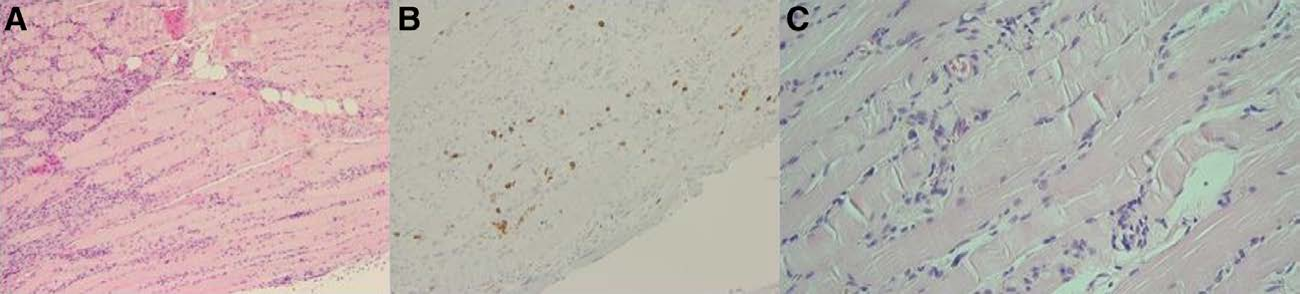
Muscle biopsy was performed on the hamstring muscle. Microscopic features show (A) endomysial and perivascular infiltration of inflammatory cells and myofiber necrosis (hematoxylin and eosin stain, ×100 magnification). (B) There are a few foci of CD8+ T cells (immunohistochemical stain for CD8, ×200 magnification). (C) Inflammatory cells are lymphocytes and macrophages (hematoxylin and eosin stain, ×400 magnification).

Initial lumbar magnetic resonance imaging (MRI) showed no definite root compression, and electrodiagnostic testing suggested right axonal sciatic neuropathy. Subsequent thigh MRI revealed diffuse hyperintensities, mainly in the hamstring muscles and partially in the adductor and vastus muscle groups of the right thigh, on fat-suppressed T2-weighted images. Contrast-enhanced fat-suppressed T1-weighted images revealed diffuse enhancement of the affected muscles (Fig. 1). Biopsy of the hamstring muscles revealed chronic inflammation and necrosis. In the inflamed tissues, endomysial and perivascular infiltration of lymphocytes and macrophages was observed, and there were a few foci of CD8+ T cell infiltration (Fig. 2). However, typical rimmed vacuoles and filamentous inclusions were not observed in myofibers. These findings can also be observed in myositis, especially in polymyositis. In the final follow-up electrodiagnostic test, myopathic potentials were observed in the vastus and adductor muscles in addition to sciatic neuropathy.

**Figure 3.**
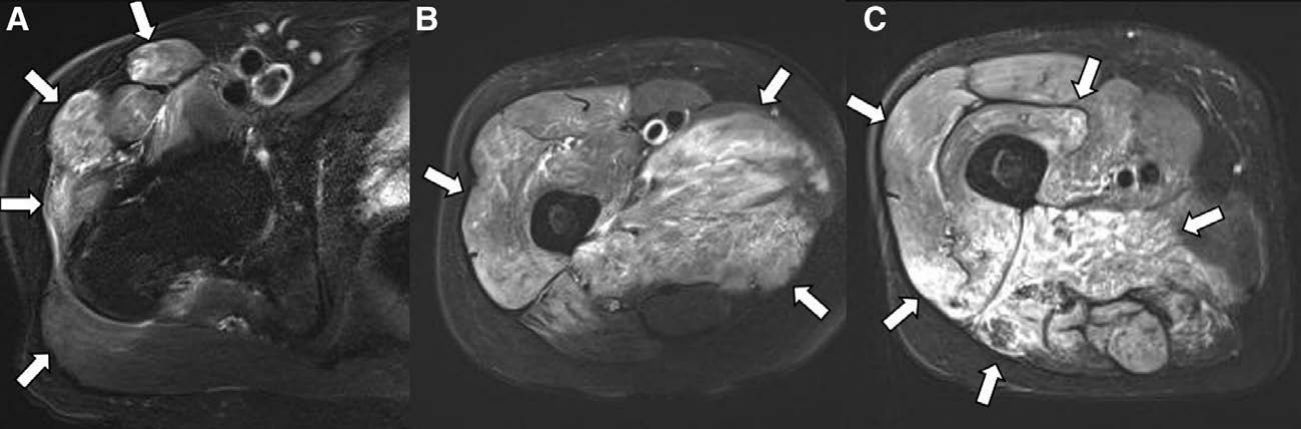
Follow-up MR images of pelvis in a patient with polyarteritis nodosa. Axial fat-suppressed T2-weighted images show (A) diffuse high signal intensity in the sartorius and gluteus muscles (tensor fascia latae, gluteus medius, and gluteus maximus) (arrows). (B) A greater extent of high signal intensity is observed in the adductor group and vastus lateralis muscles (arrows) compared with Figure 1A. (C) High signal intensity is also extended in the vastus medius and vastus intermedius muscles, while the high signal intensity in the hamstring muscles persists (arrows) compared with Figure 1A.

The patient was initially diagnosed with polymyositis combined with sciatic neuropathy and was treated with glucocorticoids. After the administration of glucocorticoids, the patient’s inflammatory markers and muscle enzymes normalized and the pain improved. However, 6 weeks later, the patient experienced newly occurring pain in his right testicle and anterior thigh. Pelvic MRI revealed that the sartorius, pectineus, gracilis, external obturator, and gluteus muscles were newly affected (Fig. 3). To differentiate lesions progressing to the proximal thigh, such as vasculitis or malignancy, computed tomography (CT) angiography and fluorodeoxyglucose-positron emission tomography/CT were performed. No occlusions or aneurysms of the visceral arteries, which can be observed in PAN, were observed on CT angiography. Positron emission tomography/CT revealed hypermetabolic lesions localized in the posterior and medial compartments of the right thigh, suggestive of localized myositis, and no malignancy was detected.

The muscle biopsy revealed myositis; however, this patient showed a negative anti-Jo-1 test, and his clinical presentation did not match the typical polymyositis pattern. Therefore, considering the symptoms of multiple systems, including the testicles, vasculitis was suspected. When ruling out other categories of vasculitis, the lack of evidence for large vessel involvement and negative results for ANCA and other autoantibodies strongly suggested that other diagnoses were unlikely. PAN was suspected based on the 2007 European Medicines Agency algorithm.^[6]^ Muscle biopsy and angiography did not reveal the typical findings of PAN; however, we made a clinical diagnosis of PAN by meeting more than 3 of the American College of Rheumatology criteria^[7]^; testicular pain, muscle weakness, mononeuropathy, HBsAg in serum, and weight loss > 4 kg. After diagnosis, the patient was treated with glucocorticoids for more than 6 months, and antiviral medication was administered to prevent HBV reactivation. The patient’s radicular pain and pain in the right anterior and posterior thighs and testicle improved, and no signs of relapse were observed. Despite the lack of improvement in the ankle motor weakness, the patient was eventually able to walk with an ankle-foot orthosis.

## 3. Discussion

In this study, we report a case of PAN that initially presented as sciatic neuropathy and myositis in the unilateral lower extremity. This case is clinically significant and unique because neurological and muscular symptoms appeared at onset without the typical involvement of PAN, and only manifested unilaterally in the lower extremity.

PAN affects multiple organs and presents with various symptoms. The kidneys, nervous system, skin, gastrointestinal tract, heart, and skeletal muscles are frequently affected by PAN. In many cases, PAN is diagnosed based on symptoms of other organs involvement before the onset of significant myopathy.^[4]^ However, in this case, there were no other organ symptoms, except for neurological and muscular symptoms. Even when testicular pain developed later, no symptoms were observed in the other organs. Furthermore, the suggestion of polymyositis through muscle biopsy made the diagnosis difficult. In addition, the initial laboratory results showed an elevation of CPK level, which is an atypical finding in PAN, making the diagnosis even more challenging.^[3,8]^

The pathogenesis of peripheral neuropathy in PAN is focal or multifocal axonal ischemic neuropathy caused by the occlusion of the vasa nervorum.^[9]^ This is consistent with the findings of axonal loss-type sciatic neuropathy observed in the patient’s electrodiagnostic tests. There are several mechanisms of muscle involvement in PAN, including ischemia due to the occlusion of blood vessels supplying the muscles and inflammatory infiltration of muscle fibers around the blood vessels.^[10]^ The latter mechanism is consistent with the perivascular infiltration findings from muscle biopsy in this patient. Furthermore, the initially affected areas in this patient were the sciatic nerve and the hamstring, adductor, and vastus muscle groups, which were supplied by the deep femoral artery. Therefore, we speculated that the patient’s initial symptoms were caused by PAN involving the vasa nervorum and muscular branches of the deep femoral artery. The areas where additional symptoms occurred thereafter, including the external obturator, pectineus, gracilis, and sartorius muscles, were also innervated by branches of the deep femoral artery. Furthermore, the gluteus muscles and testes are directly or indirectly connected to the deep femoral artery through blood vessels, including the inferior gluteal artery, which forms an anastomosis with the deep femoral artery. Based on previous cases in which the removal of the affected testicle resulted in no further involvement,^[11]^ the spread of vasculitis from the initially affected area to the surrounding areas could have accounted for the progression of PAN. However, the lack of biopsies of the suspicious arteries and testis remains a limitation, and further studies with more patients are needed.

The treatment of PAN is mainly based on corticosteroids and immunosuppressants, and the decision to administer medication is based on a five-factor score (FFS).^[12,13]^ The FFS includes the following items: central nervous system involvement, cardiomyopathy, severe gastrointestinal tract symptoms, renal failure, and high proteinuria (>1 g/d). In patients with PAN with an FFS score of 0, corticosteroids may be administered as monotherapy.^[12,13]^ In this patient, the FFS score was 0 at the time of relapse and immunosuppressants were not used because of the rapid improvement in symptoms following the readministration of corticosteroids.

Muscle biopsy may be a limitation of this case report. However, the inconsistencies between diagnosis and biopsy may be explained by several factors. First, although endomysial infiltration of CD8+ T cells is characteristic of polymyositis, it is not prominent and only a few foci of infiltration were observed, making it difficult to consider the case as typical polymyositis.^[14]^ There has also been a case report of a patient with PAN who exhibited myositis on muscle biopsy, similar to this report.^[15]^ Second, considering that the areas that showed myopathic potential on follow-up electromyography were the adductor and vastus muscles, the PAN findings may not have been reflected in the hamstring muscle biopsy. The sensitivity of muscle or nerve biopsy in symptomatic areas of PAN is approximately 60% to 70%.^[2,4,16]^ Muscle biopsy in areas where abnormal findings are observed in electromyography can increase sensitivity.^[4,17]^ If a muscle biopsy had been performed on the vastus or adductor muscles, we could have obtained findings consistent with PAN. Finally, considering a study that showed that obtaining appropriate vascular tissue through muscle biopsy is difficult because of the small size of the blood vessels,^[18,19]^ it is possible that vascular findings were not observed in this patient because muscle biopsy was performed. Calvo et al^[15]^ reported a patient with PAN who exhibited myositis on muscle biopsy but demonstrated evidence of vasculitis on nerve biopsy.

The patient initially complained of radicular pain in the lower limb in an outpatient clinic. Radicular pain is a common symptom observed during outpatient visits, and the most common cause is lumbar disc herniation.^[20]^ Other possible causes include spinal stenosis, piriformis syndrome, and nerve root tumor.^[21]^ However, radicular pain in the lower limbs can also occur because of rare conditions such as PAN, as observed in this case. Additionally, peripheral neuropathy in PAN may be responsible for the severe functional sequelae.^[9]^ Therefore, when differentiating the cause of radicular pain, especially when accompanied by severe focal muscle pain, it is important to consider PAN as a possible category.

## 4. Conclusion

This case report presents a patient diagnosed with PAN who initially presented with peripheral neuropathy and myositis in the unilateral limb without any other systemic manifestations. Early diagnosis and prompt intervention are crucial in PAN; however, in cases where only limited initial symptoms occur, diagnosis can be challenging. Therefore, when evaluating patients presenting with radicular and focal muscle pain, it is necessary to consider the possibility of PAN, as observed in this case report.

## Acknowledgments

This work was supported by the National Research Foundation of Korea (NRF) grant funded by the Government of Korea (MSIT) (No. 2022R1G1A1011574). We would like to thank Editage (www.editage.co.kr) for English language editing.

## Author contributions

**Conceptualization:** In Jun Han, Hyoseon Choi.

**Investigation:** In Jun Han, Chang Hyeon Jeong.

**Resources:** Hyoseon Choi.

**Supervision:** Hyoseon Choi.

**Visualization:** In Jun Han, Chang Hyeon Jeong.

**Writing – original draft:** In Jun Han.

**Writing – review & editing:** Hyoseon Choi.
